# Aminolysis-Based Zwitterionic Immobilization on Polyethersulfone Membranes for Enhanced Hemocompatibility: Experimental, Computational, and Ex Vivo Investigations

**DOI:** 10.3390/biomimetics9060320

**Published:** 2024-05-27

**Authors:** Arash Mollahosseini, Jumanah Bahig, Ahmed Shoker, Amira Abdelrasoul

**Affiliations:** 1 Department of Chemical and Biological Engineering, University of Saskatchewan, 57 Campus Drive, Saskatoon, SK S7N 5A9, Canada; 2 Division of Biomedical Engineering, University of Saskatchewan, 57 Campus Drive, Saskatoon, SK S7N 5A9, Canada; 3 Kinesiology, University of Saskatchewan, 87 Campus Dr, Saskatoon, SK S7N 5B, Canada; 4 Saskatchewan Transplant Program, St. Paul’s Hospital, 1702 20th Street West, Saskatoon, SK S7M 0Z9, Canada; 5 Nephrology Division, College of Medicine, University of Saskatchewan, 107 Wiggins Rd, Saskatoon, SK S7N 5E5, Canada

**Keywords:** biocompatibility, hemodialysis, membrane, polyethersulfone, zwitterion, hemocompatibility, aminolysis, computational, water stability, clinical, inflammatory biomarkers

## Abstract

Dialysis membranes are not hemocompatible with human blood, as the patients are suffering from the blood–membrane interactions’ side effects. Zwitterionic structures have shown improved hemocompatibility; however, their complicated synthesis hinders their commercialization. The goal of the study is to achieve fast functionalization for carboxybetaine and sulfobetaine zwitterionic immobilization on PES membranes while comparing the stability and the targeted hemocompatibility. The chemical modification approach is based on an aminolysis reaction. Characterization, computational simulations, and clinical analysis were conducted to study the modified membranes. Atomic force microscopy (AFM) patterns showed a lower mean roughness for carboxybetaine-modified (6.3 nm) and sulfobetaine-modified (7.7 nm) membranes compared to the neat membrane (52.61 nm). The pore size of the membranes was reduced from values above 50 nm for the neat PES to values between 2 and 50 nm for zwitterionized membranes, using Brunauer–Emmett–Teller (BET) analysis. More hydrophilic surfaces led to a growth equilibrium water content (EWC) of nearly 6% for carboxybetaine and 10% for sulfobetaine-modified membranes. Differential scanning calorimetry (DSC) measurements were 12% and 16% stable water for carboxybetaine- and sulfobetaine-modified membranes, respectively. Sulfobetaine membranes showed better compatibility with blood with respect to C5a, IL-1a, and IL-6 biomarkers. Aminolysis-based zwitterionization was found to be suitable for the improvement of hemodialysis membranes. The approach introduced in this paper could be used to modify the current dialysis membranes with minimal change in the production facilities.

## 1. Introduction

Dialysis membranes are crucial for end-stage renal disease (ESRD) patients since the chance of a kidney transplant is significantly low [1,2,3]. Polyethersulfone (PES) and polysulfone (PSF) are the most common membranes used in clinical applications and represent 93% of the market [4,5,6]. PES has a higher clearance efficiency compared to PSF [7]. Nevertheless, PES membranes still contain shortcomings, including limited hemocompatibility [1,8,9], which triggers blood activation cascades, as the rate of morbidity and mortality in ESRD patients is still unacceptably high [3,10,11,12]. Reactions between blood proteins and the incompatible PES structures trigger the functional sites of the proteins. This will consequently trigger the immune cascades in the human body, along with the inactivation and dysfunction of the proteins. Accordingly, incompatible PES membranes could trigger several side effects in ESRD patients.

Different approaches for PES membrane modifications have been investigated, ranging from non-destructive physical approaches to chemical alteration of the membrane [1,3,8,9,13,14,15,16]. Carboxybetaine (CB), sulfobetaine (SB), and phosphobetaine (PB) are the three common zwitterionic structures applied to membrane materials for hemocompatibility improvement. Zwitterions take advantage of both positive and negative charges simultaneously on one chemical moiety. This promotes better interaction with water molecules, increases the hydrophilicity (based on the general membrane surface modification mindset), limits the water mobility (based on the more recent hemocompatibility improvement theories), and creates a better protective hydration layer on the membrane surface [17,18,19]. While the efficiency of SB and PB has been approved by researchers, high production expenses and their intolerance to the harsh environment are existing concerns.

Common methodologies for zwitterionization of polymeric surfaces include layer-by-layer assembly of chemical structures, functionalization, and consequent reduction reactions, with long and complicated steps. Several SB-enhanced materials have been suggested in the literature for dialysis applications. Sulfobetaine was used as a modifier of poly(ethylene-co-vinyl alcohol) (EVOL) [20]. PSF modification using SB structures has been reported by Gu et al., which resulted in lower platelet attachment [21]. Wang et al. used SB and sodium polystyrene sulfonate (PSSNa) simultaneously to improve the hemocompatibility of PES membranes using the blending technique [22]. Polydopamine-assisted attachment of SB was reported to enhance the hemocompatibility of polylactic acid (PLA) membranes [23]. Styrene-assisted attachment of SB has been implemented to polyvinylidene fluoride (PVDF) membranes [22]. PVDF could also be functionalized with SB using a plasma environment that reacts to active fluoride sites on the membrane [24]. Click chemistry is applied to immobilize SB to cellulose membranes using a hydrophilic connector [25]. Dopamine-assisted co-deposition of SB monomers on polypropylene was also reported [26]. Most recently, it was reported that SB could fail in sterilization processes for clinical uses [27]. Ring-opening of sultone cyclic structures is one of the chemical approaches to creating SB. Dimethyl-N-(p-vinyl benzyl)-N-(3-sulfopropyl)ammonium attachment to cellulose membrane was reported by Liu et al. [28], which contained SB structure as well. Simple SB ZW structure attachment to PVDF membranes through a hydrophobic anchoring backbone was reported by Venault et al. [29]. SB was reported to be in copolymer form with sodium p-styrene sulfonate by Xiang et al. [30] for blood compatibility improvement of PSF membranes.

**Figure 1:**
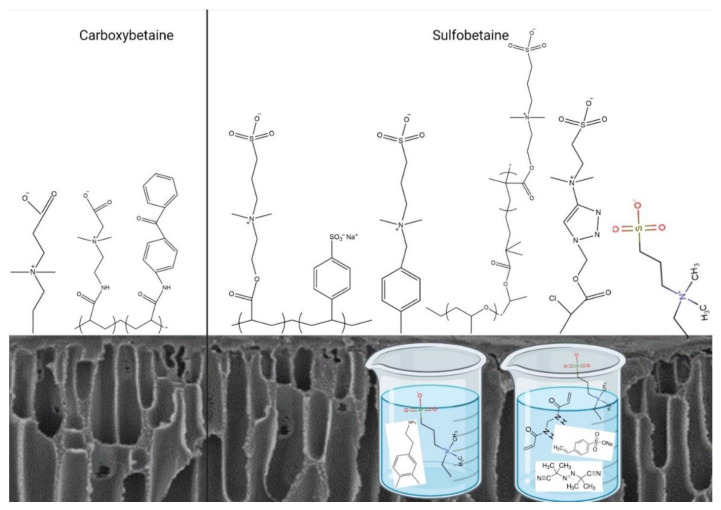
SB-assisted (right-hand side) and CB-assisted (left-hand side) hemocompatibility improvement of polymeric membranes.

CB has also been approved as a hemocompatible modification on polymeric membranes, and it has the advantage of a moderate production expense. CB has been immobilized on polyvinyl chloride (PVC) [31], PSF [32], PVDF [33], and cellulose membrane [34,35] materials. Medical-grade polyvinyl chloride (PVC) improvement with CB was reported by Lin et al. [31]. Atom transfer radical polymerization (ATRP)-assisted immobilization of CB on cellulose substrate was reported by Wang et al. [34]. Simple CB zwitterionization of PSF membranes was reported by Zhao et al. [32]. It is important to know that despite the ongoing efforts to develop zwitterionic-modified membranes, no clinical use of the products is revealed by the companies. The approach reported modifying PVDF membranes for improved hemocompatibility [33]. It is worth noting that despite the ongoing efforts to develop zwitterionic-modified membranes, there is no clinical use of the ZW products. Figure 1 shows the SB-assisted and CB-assisted hemocompatibility improvements of polymeric membranes.

Our research group has focused on interaction profiles and modification of polymeric membranes experimentally and computationally [1,9,11,36,37,38,39]. We have also reviewed the most recent theories in the field of hemocompatibility improvement [3]. As evidence, our research group has proven the addition of a common zwitterionic structure, namely, SB which contains both sulfone (negative moiety) and ammonium (positive moiety) resulted in higher hemocompatibility of PES membranes. In vitro clinical assessments reflected better immune system response and lower inflammatory biomarker secretion after the addition of zwitterionic structure [40].

Herein, we propose a facile two-step zwitterionization reaction through the functionalization method using an aminolysis reaction. This approach introduces a less time-consuming approach with fewer steps and fewer harsh chemical substances used for the surface modification of the membranes. The introduced modification approach is projected as a more efficient surface modification to be implemented on the current production lines without any major changes. The objectives of this study were as follows: (i) immobilize carboxybetaine-based and SB-based ZW coatings on the PES membrane through a facile two-step zwitterionization reaction; (ii) comprehensively assess the morphology, surface charge, and hemocompatibility of the modified membranes, clinically and experimentally; (iii) investigate the stability of zwitterionic structures and its correlation to hemocompatibility; (iv) assess the water stability on the neat and modified membranes; and (v) create a computational framework that connects the hemocompatibility to the water behavior. To the best of the authors’ knowledge, this is the first time that fast aminolysis-based zwitterionization is being used for hemodialysis membrane modification.

## 2. Experimental Methods

### 2.1. Materials

Sodium chloroacetate (98%, BCCF2967), diethylenetriamine (99%, ReagentPlus, STBJ5524), and 1,3 propane sultone (99%, BCBW3681) were purchased from Sigma-Aldrich (St. Louis, MO, USA). PES membranes were supplied by Sterlitech (ref code: 79772). Methanol (99.9%, HPLC grade, 184031) was received from Fisher chemicals. Ethanol was purchased from Commercial alcohols (Greenfield global, anhydrous, P016EAA). Deionized water was provided locally. Liquid nitrogen for differential scanning calorimetry was provided locally. Blood samples for the clinical experiments were collected from St. Paul’s Hospital dialysis center (Saskatoon, SK, Canada).

### 2.2. Experimental Approaches

**Figure 2:**
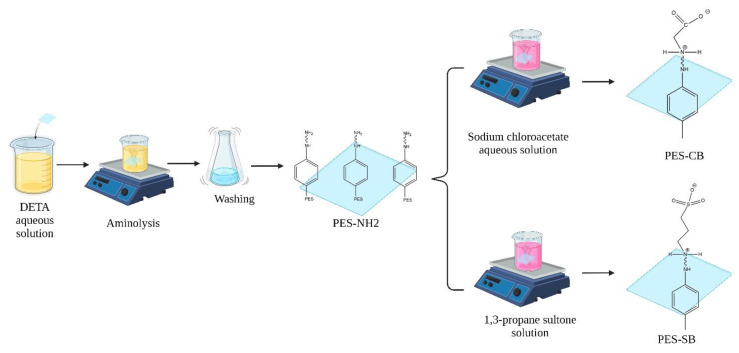
Step-by-step zwitterionization route for PES to immobilize carboxybetaine (**top**) and sulfobetaine (**bottom**).

Amine functionalization was conducted through aminolysis to induce NH2 sites on the PES membrane [41,42]. Membrane pieces (1 cm^2^) were immersed in an aqueous diethylenetriamine (DETA) solution for a certain reaction time and temperature. We conducted a comprehensive investigation to assess the influence of each condition on the area under the peak from 3200 to 3500 cm^−1^. We experimented with different temperatures (20 °C and 90 °C), reaction times (2, 24, 48 h), and DETA concentrations (wt.% in aqueous solution: 10%, 20%, 50%). Additionally, we examined the influence of UV exposure. These conditions were intended to test the robustness of the membrane. We found that room temperature (20 °C), a 2 h reaction time, and varying concentrations (10%, 20%, 50%) were not overly harsh and did not damage the membrane. Connecting the carboxyl group to the amine-containing moiety was achieved by immersing PES-NH2 membranes in an aqueous solution of sodium chloroacetate (40 g/L). (Turning amine functional groups into zwitterionic structures through the quaternary amine and sodium chloroacetate reaction has previously been reported by Zhu et al. [43]. The amine functional groups on polyethylene imine were also turned into zwitterions with sodium chloroacetate [44]). The reaction mixture was stirred at 60 °C for 12 h. For the sulfone immobilization, ring-opening of sultone was performed using 1,3 propane sultone (the zwitterionization of polymeric membrane materials through the same sultone ring opening has been previously suggested by Mi et al. [45]). The membranes in this study are named PES (neat untreated PES membranes), PES-CB (PES functionalized with CB), PES-CB-L (PES functionalized with CB, which was kept in deionized water for 10 days for stability tests), PES-SB (PES functionalized with SB), and PES-SB-L (PES functionalized with SB, which was kept in deionized water for 10 days for stability tests). Figure 2 shows a schematic illustration of the immobilization process.

### 2.3. Membrane Characterization and Analysis

#### 2.3.1. Attenuated Total Reflectance-Fourier Transmission Infrared (ATR-FTIR)

Membrane functionalization and step-by-step characterization were performed using attenuated total reflectance Fourier transmission infrared (ATR-FTIR) spectroscopy. Samples were analyzed using a Renishaw Invia Reflex Raman microscope (Renishaw Group, Gloucestershire, UK) fitted with an IlluminatIR II FTIR and a 36 × diamond attenuated total reflectance (ATR) objective (Smith’s Detection, Danbury, CT, USA), in the mid-infrared region (650–4000 cm^−1^). Membranes were cut into small (0.5 × 1 cm) pieces. Treated membranes were vacuumed and dried using a JB Platinum vacuum pump equipped with a Thermo Scientific temperature-controlled air-sealed chamber (Waltham, MA, USA). ATR-FTIR was also used for the strength of hydrogen bonding between water and the untreated PES membrane, as well as PES-SB and PES-CB. To perform this, the control and modified samples were dried in vacuum conditions for an hour. ATR-FTIR analyses were carried out at room temperature using a previously reported device equipped with a diamond ATR crystal. Deionized water (2 µL) was added using a micropipette right before the scans. The spectra were collected with a resolution of 4 cm^−1^ over 32 scans to ensure an adequate signal-to-noise ratio.

#### 2.3.2. X-Ray Photoelectron Spectroscopy (XPS)

Surface elemental analysis of the membranes in their initial neat form and functionalized state was conducted using X-ray photoelectron spectroscopy (XPS AXIS Supra, by Kratos Analytical, equipped with a 500 mm Rowland circle monochromated Al Kα (1486.6 eV) X-ray source). Membranes were vacuum-dried before scans.

#### 2.3.3. Atomic Force Microscopy (AFM)

Atomic force microscopy (AFM, nGauge, Integrated Circuit Scanning Probe Instruments (ICSPI)) was used to investigate the surface roughness patterns of the neat and modified PES membranes. The surface roughness characteristics of the samples were obtained from a 10 × 10 µm area. Parameters are recorded in the form of mean roughness (S_a_) and the root mean square (RMS) of the Z data (S_q_) [46,47]. Samples were vacuum-dried before the scans to avoid the possible effect of humidity adsorption.

#### 2.3.4. Surface Charge Measurement

The surface charge of the membrane sheets was measured using a zeta potential analyzer (Zetasizer-Nano Series, Malvern Instruments Ltd., Malvern, UK, ±0.01 mV). To perform this, members were cut to the size of the sample holder (0.5 × 0.5 cm). Membrane samples were immersed in the standard solution in a cell. The holder with the sample was immersed in 2 mM KCl solution (pH 7). The membrane’s surface charge was measured from different membrane-beam angles. The measurements were repeated three times for each measurement by the device for accuracy.

#### 2.3.5. Gravimetry

To measure the extent of modification grafting yield to the membrane, a gravimetry approach was taken. Neat membranes were dried at 40 °C for 24 h in a vacuum condition and weighted (W_1_). Zwitterionization was conducted according to the procedure reported previously. The final PES-ZW membranes were also dried at 40 °C for 24 h in a vacuum condition and weighted (W_2_). The grafting amount (GA) was calculated according to [48,49]:

(1) $$GA=\frac{W_{2}-W_{1}}{A}$$

where A is the surface area of the membrane sample. The reported results are the average values of 3 measurements.

#### 2.3.6. Equilibrium Water Content (EWC)

The equilibrium water content (EWC) experiment allows a comparison between the membranes regarding their capacity to keep water in their porous structure. The method was adopted from [50,51]. To measure the amount of EWC, membranes were cut into 1 cm × 1 cm pieces. Samples were immersed in deionized water at 30 °C for 24 h. The wet samples’ weight was measured and named W_1_. Samples were then dried at 75 °C in an oven for 24 h. The weights were measured and named W_2_. The EWC was calculated as

(2) $$EWC=\left(\frac{W_{1}-W_{2}}{W_{1}}\right)\times 100$$

#### 2.3.7. Differential Scanning Calorimetry (DSC)

The methodology to study the hydration state of polymers using the DSC was adopted from [52]. The membrane samples in neat and modified form were cut to a size that could fit in the DSC pan. EWC was measured according to Section 2.3.6. DSC experiments were performed using the Q2000 TA instruments (New Castle, DE, USA). The hydrated samples were put in an aluminum pan, and the pan was sealed with an auto sealer. The pans were cooled to −60 °C at a rate of 5 °C/min and held at −60 °C for 5 min. The samples were then heated for the ice-to-water cycle at the same rate to 40 °C. The DSC heating profile of PES was used as the control. The DSC device was used to measure the amount of free water. Based on the equations introduced in [53,54], the amount of stable water (non-freezable water) on the surface is calculated as follows:

(3) $$w_{c}=\omega_{freezable}+\omega_{non-freezable}=\frac{∆H_{freezable}}{∆H_{Bulk}}\times 100+\omega_{non-freezable}$$

where $∆H_{Bulk}$ is equal to 355 J/g, and $∆H_{freezable}$ can be obtained from the thermograms of the DSC.

#### 2.3.8. Brunauer–Emmett–Teller (BET)

Brunauer–Emmett–Teller (BET) and Barret–Joyner–Halenda (BJH) were used for the specific surface area (SA), pore volume, and average pore size measurements of the membrane samples [55]. To conduct the measurements, membrane samples were initially degassed under vacuum conditions. Since the zwitterionic structures could decompose easily at higher temperatures [56], membranes were degassed at 50 °C for 3 h [57]. A Quantachrome Instrument (NOVA touch LX^2^) (Boynton Beach, FL, USA) operated with inert nitrogen gas was used for the experiments.

### 2.4. Computational Studies

#### 2.4.1. Force Field and Software

The interatomic behaviors of the structures were defined using Dreiding potential [58]. Dreiding forcefield is an old potential, yet the authors were trying to model both polymeric and biological structures, which could be covered by the same forcefield. We have published other papers using the same force field [38,59], and we have published one with the same methodology, which was in agreement with our molecular docking simulations as well [37]. Several other papers are using the force field, despite its age [60,61,62,63]. Table 1, Table 2 and Table 3 introduce the parameters used for the simulation. Sandia National Laboratories created the Large Scale Atomic/Molecular Dynamics Parallel Simulator (LAMMPS) software (GPLv2 license) for MD simulations [64]. Simulations were conducted with periodic boundary conditions for all the dimensions using the NVE ensemble, with a time step of 0.001 ps and a temperature of 298 K controlled by the Langevin thermostat. VMD software (version 1.9.4) was used to create the visualizations [65]. The polymeric chains were built and minimized using Avogadro software (version 1.99.0) [66]. Protein models were received from the protein data bank. Simulation box preparation and packing were performed using PACKMOL software [67].

#### 2.4.2. Structures and Simulations

The polymeric structures simulated in this study are introduced in Table 4. Hydrogen bonding assessments were performed by locating each of the structures in Table 4 in a hydration box with 60 water molecules. The number and the energy of the hydrogen bonding in each simulation were assessed. The mobility of the hydrated structures was also studied by creating a membrane model (including 10 polymeric chains) covered by two layers of water molecules on the top and bottom. Details of the simulations are listed in Table 5. The mobility of the systems was calculated by the mobility function of water molecules according to Equation (3) [68]:

(4) $$C\left(∆t,Z\right)=\frac{\langle {\left(r_{i}\left(t_{0}+∆t\right)-r_{i}\left(t_{0}\right)\right)}^{2}z\rangle }{MSD\;of\;molecules}$$

where the term in 〈 〉 is the average mean square displacement ensemble, and $r_{i}$ is the location of molecules.

### 2.5. Hemocompatibility Measurements

Clinical in vitro assessments were performed to measure the extent of several biomarkers in the bloodstream [57]. To do so, uremic blood from hemodialysis patients 60 years of age or older at Saint Paul Hospital (Saskatoon, SK, Canada) was collected according to the ethics protocols for data measurements and model development [63,64]. The research population consisted of two groups: uremic patients (n = 5) and normal controls (n = 5). The patients had a BMI of ≥27 kg/m^2^ and were without additional medical conditions such as diabetes, hypertension, or peripheral vascular disease. The targeted biomarkers are listed as: interleukin-1 (IL-1), interleukin-1β (IL-1b), interleukin-6 (IL-6), properdin, von Willebrand factor (vWF), and complement components C5a and C5b-9. The procedures, model developed, and statistical analyses are described in detail in our recent studies [69,70].

The surface charge of the treated membranes, in addition to the in vitro clinical inflammatory biomarkers, has been tested after keeping the PES-CB and PES-SB samples in deionized water for 10 days. Storing the modified membranes in deionized water for a specified period is an approved approach for assessing the stability of ZW on the membrane surface [71].

## 3. Results and Discussion

### 3.1. Characterization of Neat and Functionalized PES Membranes

As the first step of PES membrane modification, amine functionalization was performed. The common technique to immobilize amine groups on PES is through HNO_3_ and H_2_SO_4_ mixture acid washing followed by a reduction reaction in Na_2_S_2_O_4_ solution [72,73]. While the technique is properly capable of immobilizing amine functional groups on PES membranes, long reaction times, drying periods, and neutralization procedures make the approach less favorable for fast production. Aminolysis could be used as a substitute method of amine functionalization in only one step of membrane soaking [41,42]. Common aminolysis reaction conditions are reported as 10 wt.% of amine-containing monomer (DETA or dipropyltriamine (DPTA) aqueous solution), 48 h of reaction time, and at 90 °C [41,42]. We have assessed the initial reported conditions. We also assessed if amine functionalization could be performed at room temperature and within a shorter reaction time.

**Figure 3:**
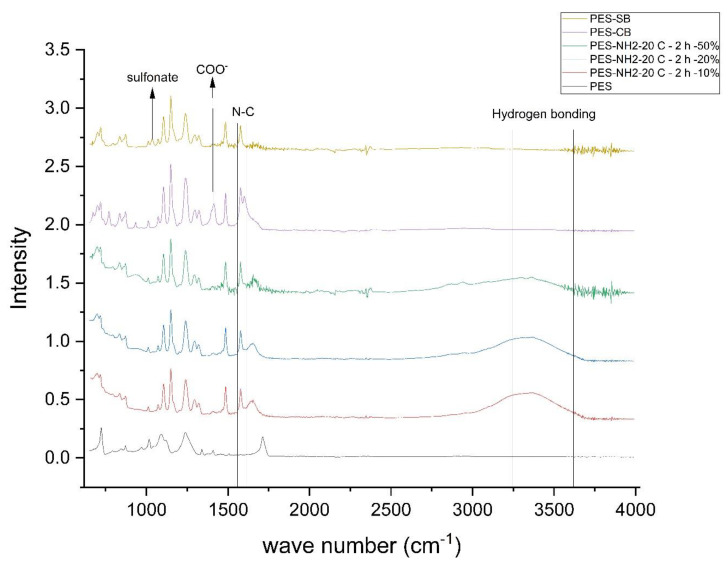
ATR-FTIR characterization of the PES neat membrane, amine functionalized PES membranes at different DETA monomer concentrations, and carboxybetaine- and sulfobetaine-modified membranes.

Figure 3 reflects the spectra for PES in its neat and amine-functionalized state. The characteristic peaks for the PES membrane are the weak asymmetric stretching vibration peak around 1340 and the symmetric vibration peak at 1134 cm^−1^ [38]. The sulfone characteristic ring could also be located in a different location (stretching vibrations of 1242 cm^−1^ and 1124 cm^−1^ for the PSF membrane [74]). Aminolysis resulted in the formation of a weak peak around 1294 cm^−1^, which represents the stretching vibration band of C-N, confirming that nitrogen is attached to the carbon backbone of the PES [38]. Common IR amine peaks are reported to be between the wave numbers of 3200 and 3500 cm^−1^ [75]. Previous reports of aminolysis performed on PES also suggest the same range for the desired amine [76]. Accordingly, the wide peak in this region reflects the successful amine functionalization of PES. After the functionalization of PES by the aminolysis reaction, the stretching peak for NH appeared near 1636 (secondary amine) and between 3300 and 3500 cm^−1^ The two bands of NH appeared at 3390.53 and 3416.13 cm^−1^, and the deformation band of NH_2_ was at 1618.92 cm^−1^ [77]. The obvious peak between 2300 and 2400 cm^−1^ represents the C-N bond, which approves the chemical attachment of amine functional groups [78]. In addition, the peak that appeared between 2300 and 2400 cm^−1^ represents the C-N bond, which approves the chemical attachment of amine functional groups [78].

**Figure 4:**
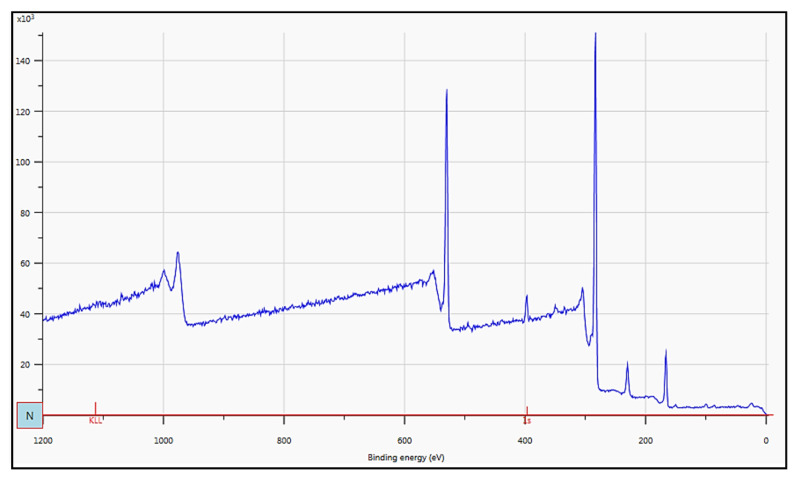
XPS analysis of amine-functionalized PES membranes through an aminolysis reaction.

The effect of DETA concentration was studied at a fixed reaction time and temperature. As depicted in Figure 3, the aminolysis reaction resulted in more pronounced peaks in the range of 3200 to 3500, which means more NH structures are created on PES membranes. DETA-assisted amine functionalization of other membranes has also been reported by Al-Shaedi et al. [79]. Another IR peak is around 660–670 cm^−1^. As presented in Figure 3, the peaks in the announced range are also intensified at the 50% concentration of DETA. The C-N peak around 2350 cm^−1^ is also intensified for the PES membrane functionalized with 50% DETA solution. Figure 4 reflects the XPS of the PES membrane functionalized with 50% DETA solution. The peak near 400 eV reflects the existence of the nitrogen-containing structure in the functionalized membrane. Accordingly, aminolysis with moderate temperature and reaction time and a higher concentration of DETA monomers could successfully result in the amine functionalization of PES membranes.

After proving the aminolysis step, we assessed the functionalization of SB and CB, as shown in Figure 3. ATR-FTIR assessment of the PES-CB membrane sample reveals the formation of the zwitterionic structure by the simultaneous presence of two peaks. The first peak near 1640 cm^−1^ was attributed to quaternary nitrogen [80]. The COO^−^ was confirmed by the peak at 1380 cm^−1^ [81].

The chemical footprint of the PES-SB reflects the comparison between PES-NH2 and PES-SB. Since PES has sulfone structure, the peak related to the S=O group exists at 1160 cm^−1^. However, the small peak between 1030 and 1050 cm^−1^ reflects the sulfonate functional group as the end moiety of the SB ZW structure [82]. The peak near 1640 cm^−1^ is attributed to quaternary nitrogen [80]. The obvious peak between 2300 and 2400 cm^−1^ is also mentioned to represent the C-N bond which approves the chemical attachment of amine functional groups [78].

**Figure 5:**
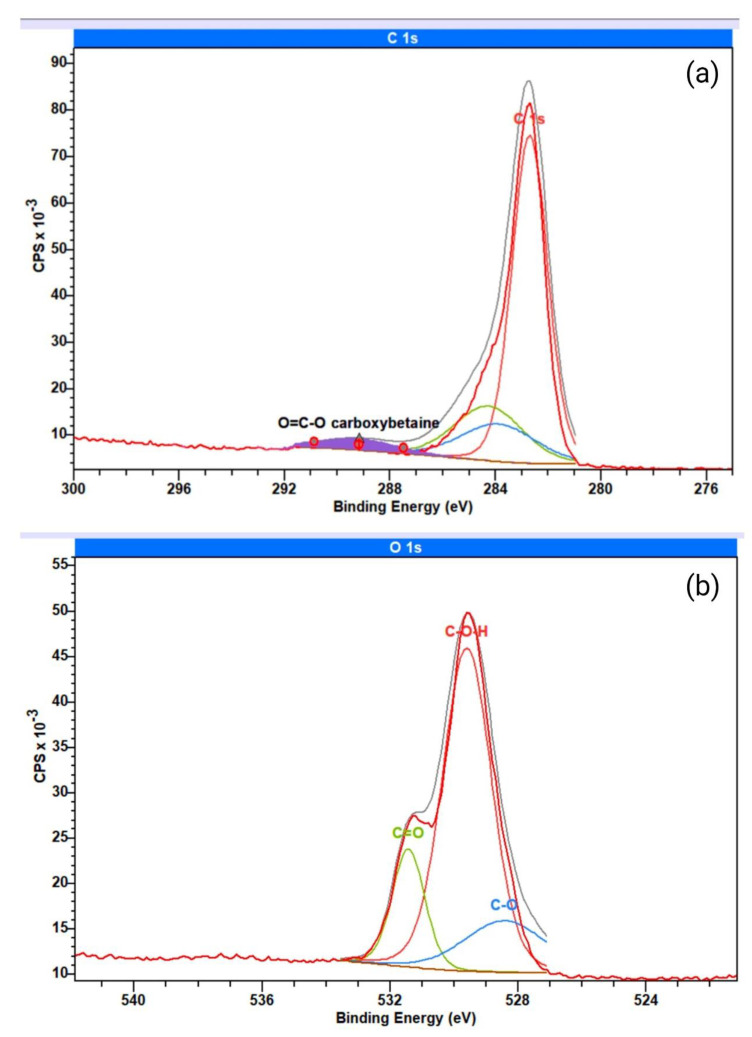
High-resolution XPS of PES-CB membrane: (**a**) C1s; (**b**) O1s.

To further assess the final chemical structure of PES-CB, we made a high-resolution C1s XPS scan. Figure 5a reflects the XPS spectra of PES-CB. The peaks around about 284.8 and 285.4 eV are attributed to C-H (as well as C-C) and C=O structures. The broad peak at 289.5 could be attributed to the O=C-O of the CB moiety of the zwitterionic structure [34].

The key steps taken with ATR_FTIR analysis for the surface modification of PES with CB are locating amine sites on the PES using aminolysis proven by the amine and hydrogen bonding peaks around 1650 cm^−1^ and after 2300 cm^−1^, respectively, and locating the final negative carboxyl moiety proven by the peak at 1380 cm^−1^. Similar assessment on the implication of carboxybetaine on porous substrates reflects a similar chemical footprint of symmetric stretching bond for COO^−^ from 1380 cm^−1^ to 1454 cm^−1^, along with overlap of the peaks with symmetric and asymmetric peaks of -CH_3_ [83].

The path for identifying successful implementation of SB on the membrane surface starts with locating the amine at 1650 cm^−1^, and adding a sulfonate functional group at 1030 cm^−1^ and 1050 cm^−1^. The wave number is also in agreement with the location of the sulfonate of sulfobetaine methacrylate at 1033 cm^−1^ [83].

Figure 5b reflects the oxygen assessment of the PES-CB membranes. The appearance of oxygen peaks proves the CB-negative moiety. Zwitterionization of the PES membrane resulted in a broadened oxygen peak, which could be split in binding energies and could be divided into two extra split peaks rather than the C-O-H peak. The two peaks, 528.5 eV for the C-O bond and 531.5 eV for C=O, represent [O-C=O] and [O-C=O^−^] structures in the CB [84].

**Figure 6:**
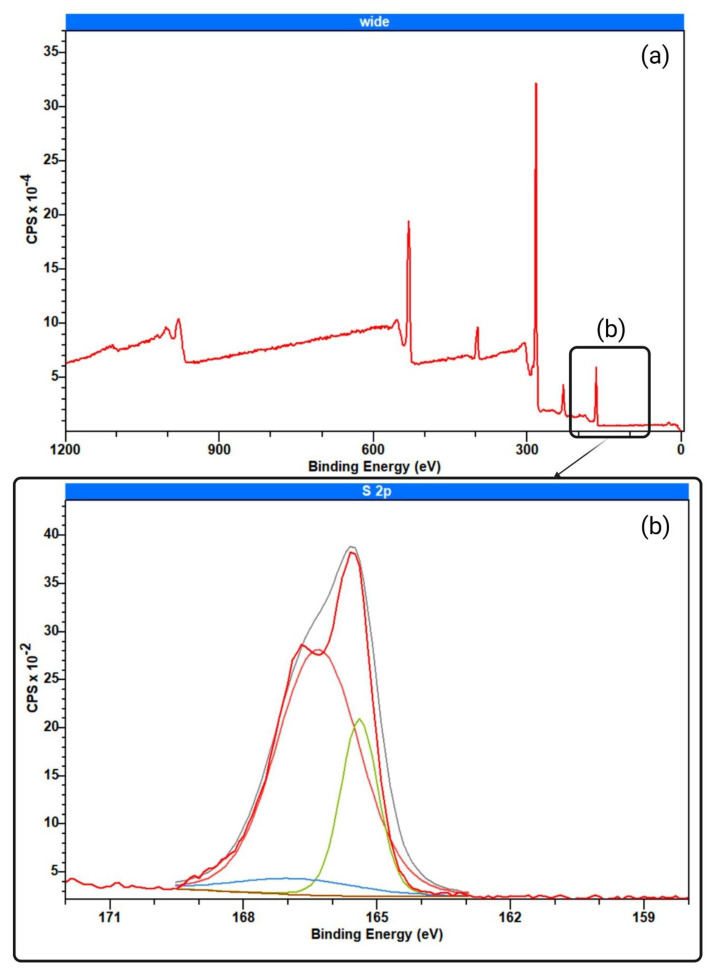

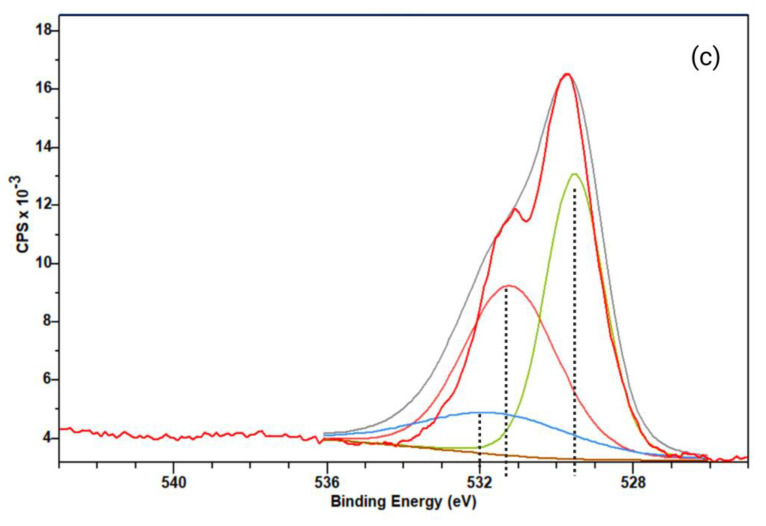
XPS analysis of sulfobetaine-functionalized PES membranes: (**a**) wide-angle scan; (**b**) scan of S 2p and (**c**) O 1s.

XPS analysis of the PES-SB membrane is presented in Figure 6. The wide-angle scan of the membrane is shown in Figure 6a. The peak at 167 eV (S 2p) represents the sulfur in the prepared membranes (Figure 6b). The deformed S 2p peak for the PES-SB membrane is due to the addition of the SO_3_^−^ moiety of the ZW structure, which could be divided into three peaks similar to previously reported characteristics of SBMA [85]. O 1s analysis of PES-SB also shows the peak of SO_3_^−^ at 532 eV (Figure 6c) [86].

Table 6 reflects the elemental analysis of the neat and modified membranes through XPS spectroscopy. The nitrogen percentage was less than 1% in the PES sample, which could be due to the trace amount of nitrogen trapped in the microporous structure of the membrane surface or to probable pollution on the surface. Aminolysis resulted in a higher amount of nitrogen on the membrane surface, from 0.92% to 4.26%. The PES membrane had 5.90% of sulfur. Surface coating of the amine and carboxyl functional groups resulted in the addition of a top layer with a diluted amount of sulfur in the case of PES-NH2 and PES-CB (5.21% and 4.90%, respectively). However, the sulfonate group located on the PES-NH2 membrane to form PES-SB resulted in a higher content of sulfur on the top surface of the membrane (6.61%). The highest carbon and oxygen contents (75.39% and 16.54%) on the modified membrane surfaces belong to PES-CB, which has the highest content of the carboxyl functional group.

### 3.2. Hydrogen Bonding Assessment

There are different approaches to identifying the stability of water molecules in a polymeric matrix. We have previously reported spectroscopy-based techniques such as IR-spectroscopy and NMR for qualitative and DSC for the quantitative assessment of the three types of water (namely, free water, intermediate water, and non-freezing water [3]). The advantage of IR spectroscopy over other techniques is its accessibility and low cost. Previous research efforts over the IR-assisted water behavior study reflected two regions for water molecules. The broad peak at around 3200 cm^−1^ is related to the strong hydrogen bonding of water molecules, while the broad peak around 3500 cm^−1^ is of the less strong hydrogen bonds. In other words, stronger hydrogen bonds tend to have lower wave numbers [87,88]. It is important to note that the adjustment of the humidity as well as the amount of water exposed to the membrane material affects the shape of the peaks [89]. More importantly, the experiment could be time-sensitive, depending on the membrane material used [90].

**Figure 7:**
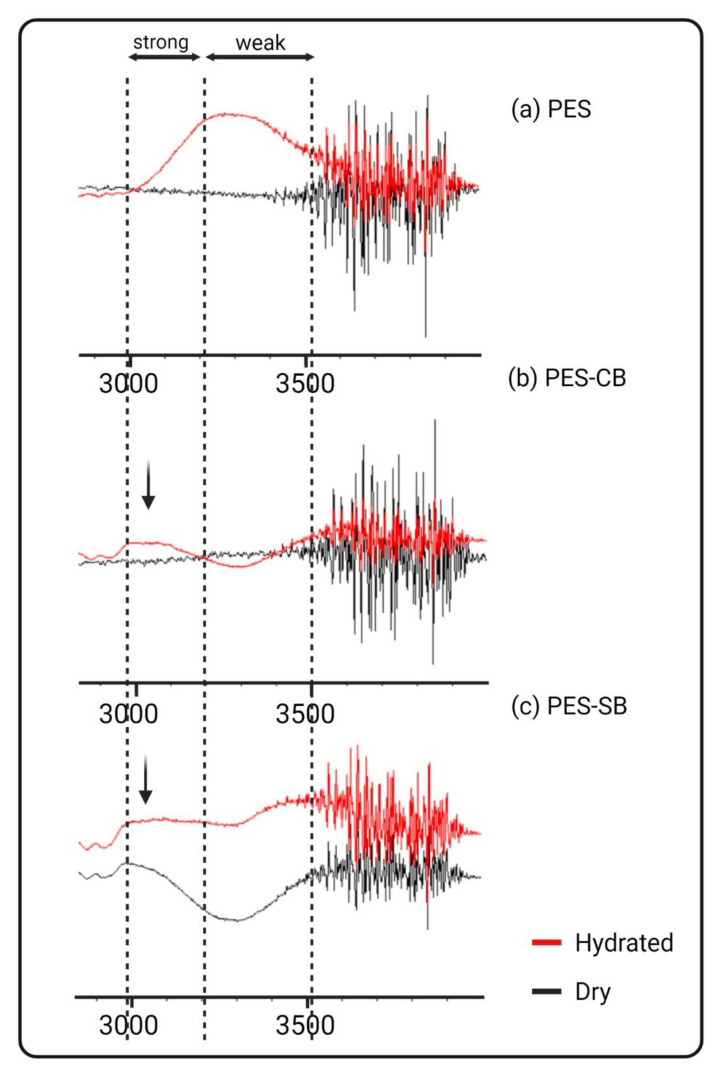
Water interaction with different membrane surfaces: (**a**) PES, (**b**) PES-CB, and (**c**) PES-SB.

Figure 7 reflects the ATR-FTIR spectra of the PES control sample (Figure 7a), PES-CB (Figure 7b), and PES-SB (Figure 7c). The black spectra are for the dry samples, and the red ones are hydrated with the measured amount of water. For the PES sample, most of the hydrogen bonding is leaning toward the weaker region (3500 cm^−1^). The PES-CB has most of the hydrogen bonds between (3000 and 3200 cm^−1^). The black PES-SB spectra in dry mode have the same peak as the hydrated PES-CB, in the strong hydrogen bond region. This means that even in the vacuum chamber, the hydration layer, which is strongly associated with the surface, could not be removed. After adding water, the peak in the 3000 and 3200 cm^−1^ became broader, which reflects a higher quantity of strong hydrogen bonding. On the other hand, weaker hydrogen bonds at higher frequencies were also increased.

### 3.3. Surface Roughness

**Figure 8:**
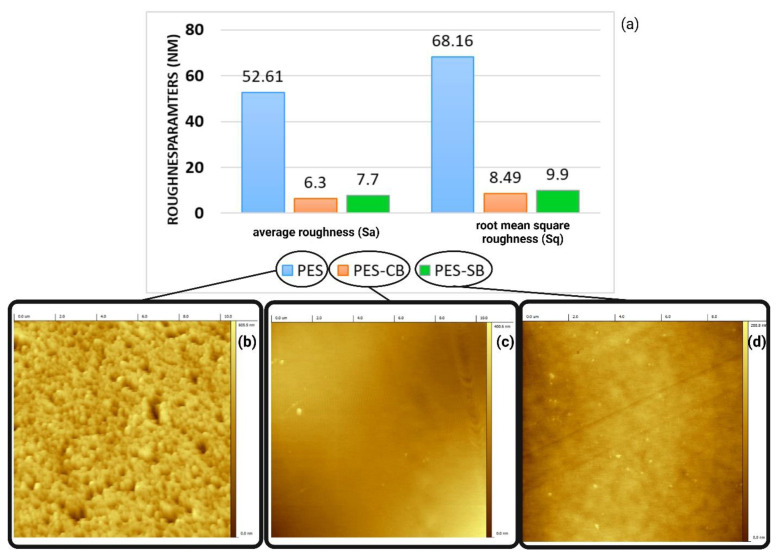
AFM scan results of neat and surface-treated membranes: (**a**) roughness parameters; (**b**) surface topology of PES membrane; (**c**) surface topology of PES-CB; (**d**) surface topology of PES-SB.

Figure 8 reflects the scanned surface of the membrane using the AFM microscope and the roughness parameters. Figure 8 reflects the values in nm for Sa and Sq. Figure 8b,c,d reflect the scans for neat PES, modified PES-CB and PES-SB membranes, respectively. Both roughness parameters have significantly dropped after the surface modification.

The mean roughness significantly decreased from 52.61 nm for neat PES to 6.3 nm for CB (88% decrease) and 7.7 nm(85% decrease) for SB. RMS roughness declined from 68.16 nm for neat PES to 8.49 nm (87% decrease) for CB and 9.9 nm(85% decrease) for SB. Figure 8d reflects the PES-SB membrane. As it could be seen visually (as well as quantitative data in Figure 8a), CB addition to the PES membrane resulted in lower roughness. Depending on the nature of the modification layer’s chemical structure and the geometry of the material, the ZW top layer could fill the pores of the membrane to an extent and reduce the roughness [91]. Modifications that reduce the roughness of the surface could result in a higher degree of hemocompatibility due to a lower hemolysis ratio [92,93]. According to our previous investigations, we have proved that lowering the roughness could enhance hemocompatibility [69,70].

Commonly, smoother surfaces (less rough, lower values for S_a_ and S_q_) are known to act in favor of hemocompatibility. This is due to the fact that rough surfaces would rapture the blood cells, and this would ultimately result in a higher hemolysis factor (lower hemocompatibility). Another point to consider is that the bloodstream is loaded with more than 3700 types of proteins, which makes the membrane prone to fouling intensively. Accordingly, a less tough surface for the modified surface could result in lower plasma protein fouling. Provocation of the plasma proteins is the initial stage for their activation, which consequently results in complement, thrombogenesis and coagulation, inflammation, and leukocyte activation [1,2,3].

### 3.4. Surface Charge Analysis

**Figure 9:**
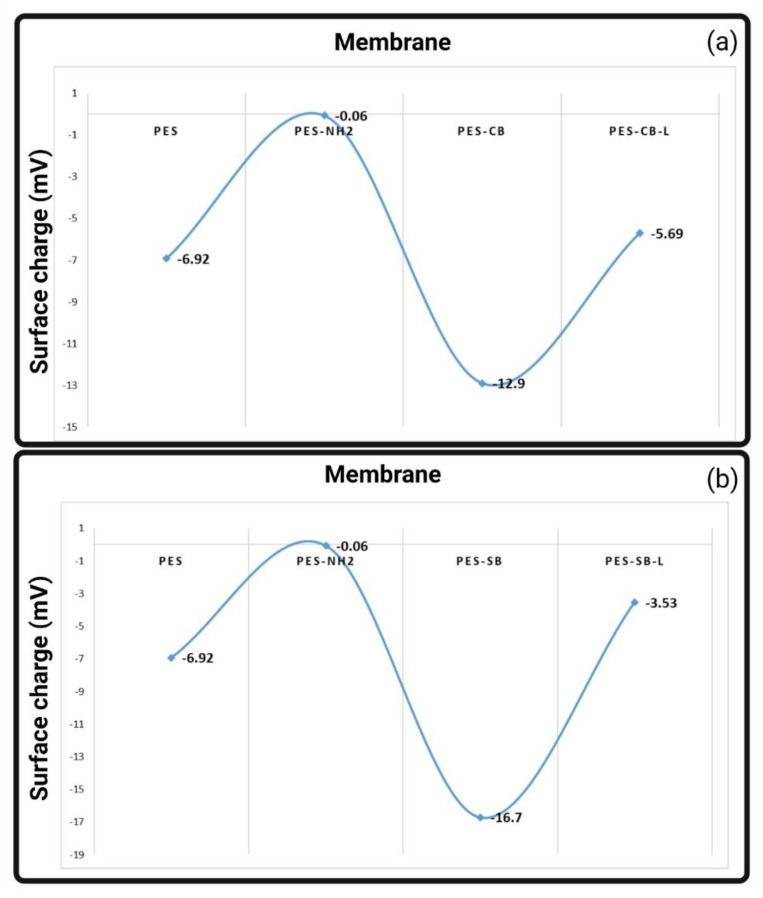
Surface charge variation of PES neat and functionalized membrane; (**a**) surface charge trend of PES-CB; (**b**) surface charge trend of PES-SB.

Surface charge is a crucial characteristic for interfacial interaction assessment, fouling, biocompatibility, and hemocompatibility. A common understanding of dialysis membranes is used to infer that more hydrophilic groups, such as carboxyl and hydroxyl functional groups, would result in better hemocompatibility [1]. However, negative surface charges were reported to trigger the kallikrein/kinin system and blood coagulation cascade toward an incompatible membrane-blood interaction [94]. The emergence of the third generation of dialysis membrane modifiers reflected that not all negatively charged surfaces are blood-friendly, and a better hemocompatible surface would be obtained by electroneutral surfaces [1,9]. Accordingly, an ideal modification to our PES membranes would have been an electroneutral CB. As reflected in Figure 9a, the surface charge of the neat PES membrane was −6.92 mV. The addition of amine as the positive block of the zwitterion increased the membrane’s surface charge to a value near 0 mV. The addition of the carboxyl final structure to form the zwitterion decreased the charge to a value of −12.9 mV. The last membrane sample, named PES-CB-L, showed a loss of surface charge to a value between neat PES and PES-NH2. The increase of the surface charge after aminolysis and its drop to a negative value, besides the FTIR and XPS characterizations, reflect that we have successfully immobilized the CB structure on the surface. However, since the number of amino and carboxyl functional groups was not optimized, more negative functional groups were located on the surface. Keeping the membranes in deionized water resulted in the loss of loosely attached negative charges, and the final product, i.e., PES-CB-L, had a less charged surface. Figure 9b reflects the same trend for the SB-modified PES membrane. Compared to PES-CB, PES-SB was more negatively charged. Moreover, the PES-SB-L lost more negative charge in comparison with PES-CB-L. Accordingly, SB-modified membranes experienced a wider range. Since PES-CB-L experienced a less intense charge loss, we conclude that CB has a more stable chemical structure on the PES membrane. While a similar trend for the two zwitterionic structures was expected, the difference in charge range could be due to the different nature of the chemicals used for the final negative block of the zwitterionic structure. The final negative charge is not favorable for an electroneutral ZW structure, as it was previously explained that negative charges trigger coagulation. However, it should be noted that the negative charge here is coupled with the positive moiety of quaternary ammonium within the structure of ZW. The water interaction of ZW moieties creates a more efficient hydration layer to protect blood proteins from reacting with the membrane. Accordingly, a more blood-compatible profile is expected.

### 3.5. Gravimetry-Assisted Grafting Amount Measurement

Table 7 reflects the weight variation of PES membrane samples after being modified with zwitterionic structures. The amount of modification adhered to the PES membrane after SB modification was slightly higher than CB. The grafting amount for SB modification was 0.05 mg higher than CB. However, considering the error range of the measurements, both modifications are within the same range. Other zwitterionization methodologies have also reported a range of a few hundred µg grafting weight change, which agrees with the measurements presented here [82].

### 3.6. Equilibrium Water Content (EWC)

Table 8 reflects the EWC of the neat and modified PES membranes, according to the method reported in the methods section. PES was able to absorb 43.13% more water. The EWC value of an unmodified PES membrane could stand between 25% and 72% depending on the nature of the surfactants, pore formers, and solvents used for the fabrication of the membrane (the hydrophilic nature of PES allows it to absorb water easily due to its ether and sulfone functional groups) [95,96]. The addition of CB to PES resulted in a nearly 6% increase in EWC. PES-SB had 9% more EWC compared to PES. It is common to observe an increase in the amount of EWC when hydrophilic modifiers are added to the structure of the membrane [95].

### 3.7. Stable Non-Freezable Water Content

**Figure 10:**
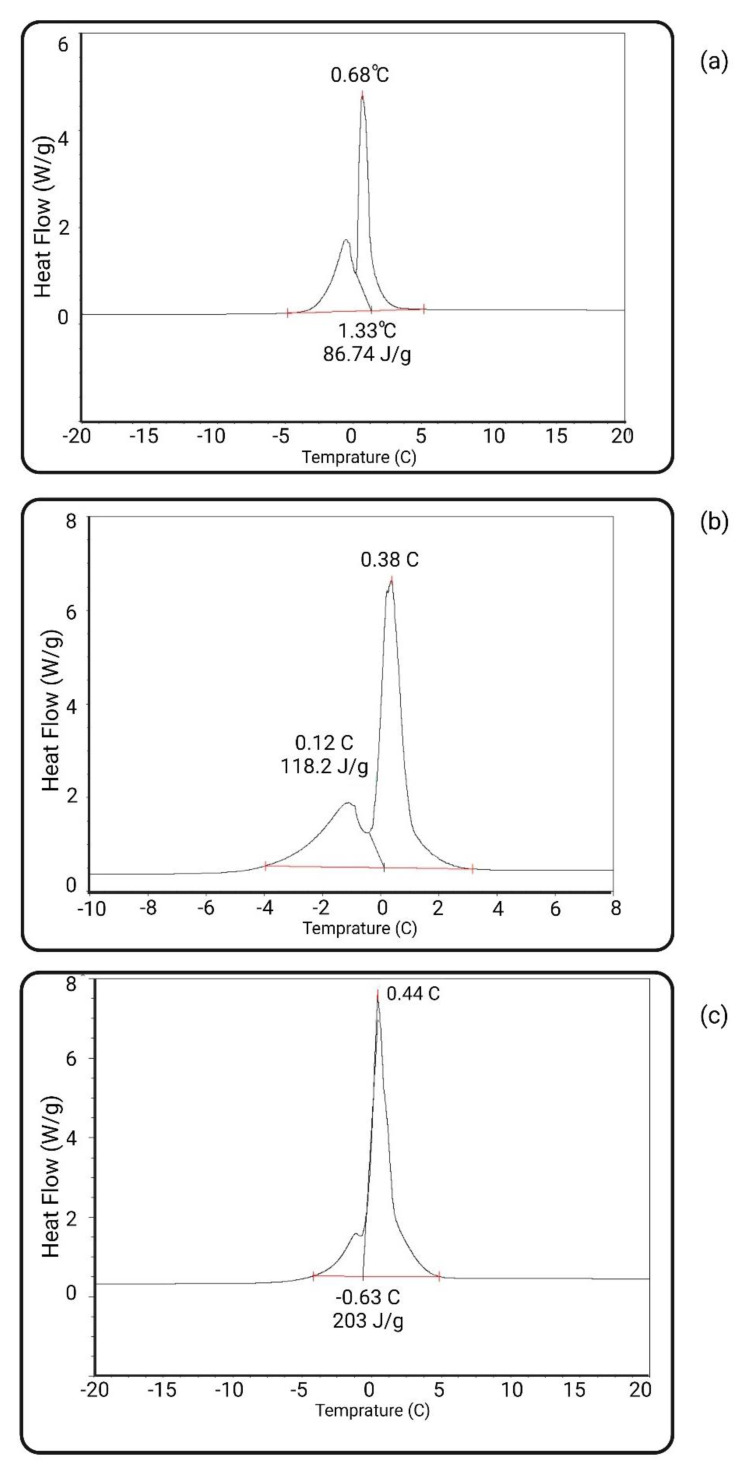
DSC peak of neat and PES-ZW membranes: (**a**) neat PES; (**b**) PES-CB; (**c**) PES-SB.

The stable water content was measured using a DSC device according to Equation (3). Table 9 reflects the amount of non-freezable water for the neat, PES-CB, and PES-SB membranes. Figure 10 reflects the DSC scans of the membranes. PES-SB had the highest percentage (16.28) of non-freezable water in comparison with the two other membranes. PES-CB membrane also had 12.85% of the stable water. The results of the stable, non-freezable water amount are a function of the zwitterionziation approach and the final concentration of the modification layer on the membrane. It is also a function of the chemical structure placed on the membrane surface as well. Since the chemical approach to attaching CB and SB was different regarding the last negative moiety, the difference between the stable water was expected.

Figure 10 represents the peaks for the amount of water that could freeze. The temperature and enthalpy of the freezing are reflected on the peaks. As it is reflected, the average temperature of the freezing (for the overall peak) decreases as the modifications are added to the membrane. T_freezing_ was 1.33 °C, 0.12 °C, and −0.63 °C for PES, PES-CB, and PES-SB, respectively. This could be interpreted as the higher association of freezable water to the surface, which prevents the water molecules from forming ice crystals at the normal temperature.

### 3.8. Pore Size Assessments

**Figure 11:**
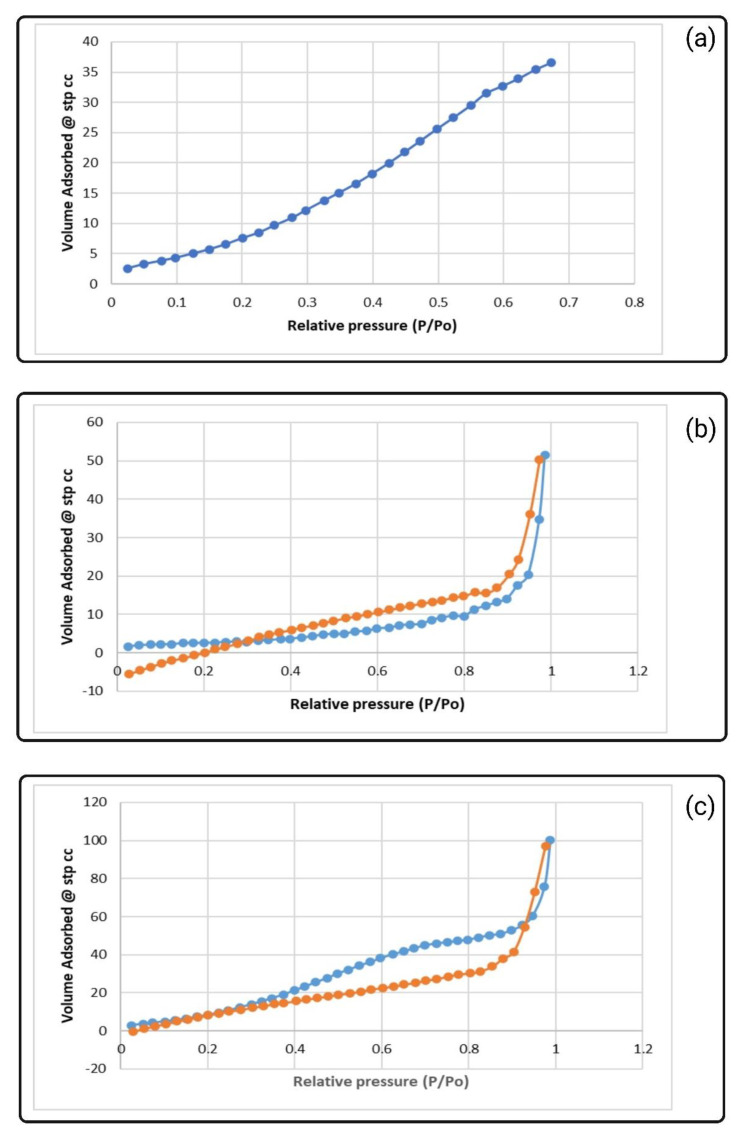
Nitrogen adsorption–desorption isotherms for neat and zwitterionized membranes: (**a**) PES; (**b**) PES-CB; and (**c**) PES-SB.

Table 10 reflects the specific surface, pore volume, and pore diameter of the neat and zwitterionized PES membranes measured using a BET device. Compared to the PES membrane, the specific surface area and pore volume of the zwitterionized membranes are improved. The surface area was increased more for PES-CB (93.50 m^2^/g), while the pore volume was increased to a greater extent for PES-CB (0.16 cm^3^/g, almost 3 folds higher than the pore volume of PES membrane). Figure 11 reflects the BET isotherms of the samples. Nitrogen adsorption and desorption for the PES neat membrane were similar. Accordingly, PES had a microporous structure with an average pore size greater than 50 nm. After zwitterionization, hysteresis forms were created in the isotherms (type IV), which reflect a different behavior in the adsorption and desorption of the nitrogen gas. This is due to the smaller pore formation within the membrane surface, which resulted in a different pattern of nitrogen desorption. Accordingly, based on the form of the BET isotherms, an average pore size of 2 to 50 nm is considered for both PES-ZW membranes.

### 3.9. Hydrogen Bonding Simulation

**Figure 12:**
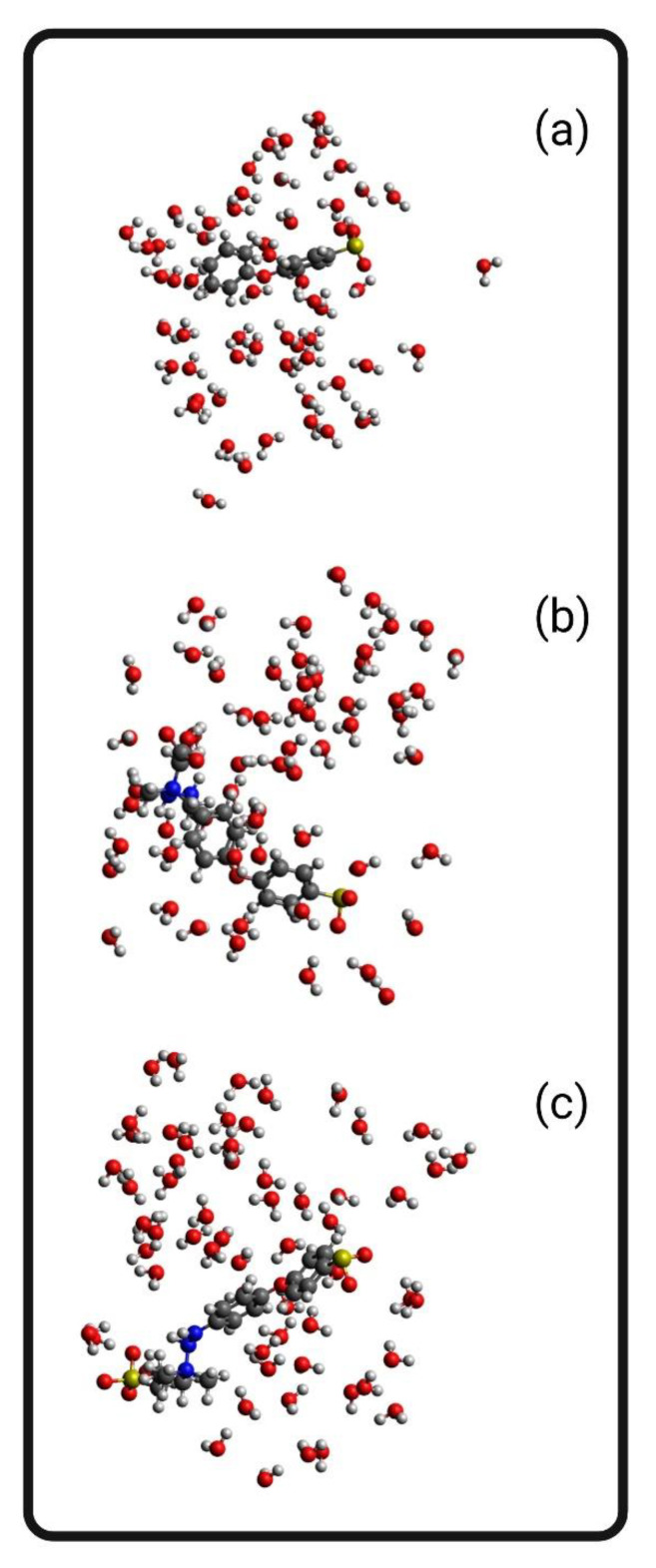
Mobility simulations: (**a**) PES, (**b**) PES-CB, and (**c**) PES-SB.

Figure 12 reflects the simulation captures of the hydration boxes for neat and modified structures. Table 11 reflects the number and intensity of hydrogen bonding between the modeled polymers and water molecules in a simulated hydration box. As discussed in phase 3, the high intensity of hydrogen bonding for PES, which could originate from the oxygens of the sulfone and ether functional groups, has the potential to interact with the functional groups of the membranes. The addition of PVP resulted in a higher number of hydrogen bonds and a lower intensity of hydrogen bonding. Accordingly, lower and more moderate values for the E/n parameter could result in higher hemocompatibility. PES-CB and PES-SB both had moderate hydrogen bonding energies (−1.45 and −0.03 kcal/mol, respectively) in comparison with the PES membrane (−14.37 kcal/mol). PES-CB simulation resulted in 11 hydrogen bonds, while PES-SB resulted in only 2 hydrogen bonds, despite the expectations. This might be due to the random location of water molecules in the simulation or the size of the simulation box. However, the E/n parameter for both PES-CB and PES-SB was less than the PES structure. A more moderate profile of hydrogen bonding interactions could be interpreted as a less intense tendency to move water molecules. Several incompatible surfaces trigger the hydration layer around proteins by removing the water molecules and interacting with the amino acid active sites [3]. More frequent hydrogen bonds between water molecules and the membrane surface with an overall lower E/n value could reflect a more stable protective hydration layer formed on the membrane surface without the ability to remove hydration layers around the proteins. Accordingly, the simulation reflects the higher hemocompatibility of the PES-CB and PES-SB membranes.

### 3.10. Mobility Simulation

**Figure 13:**
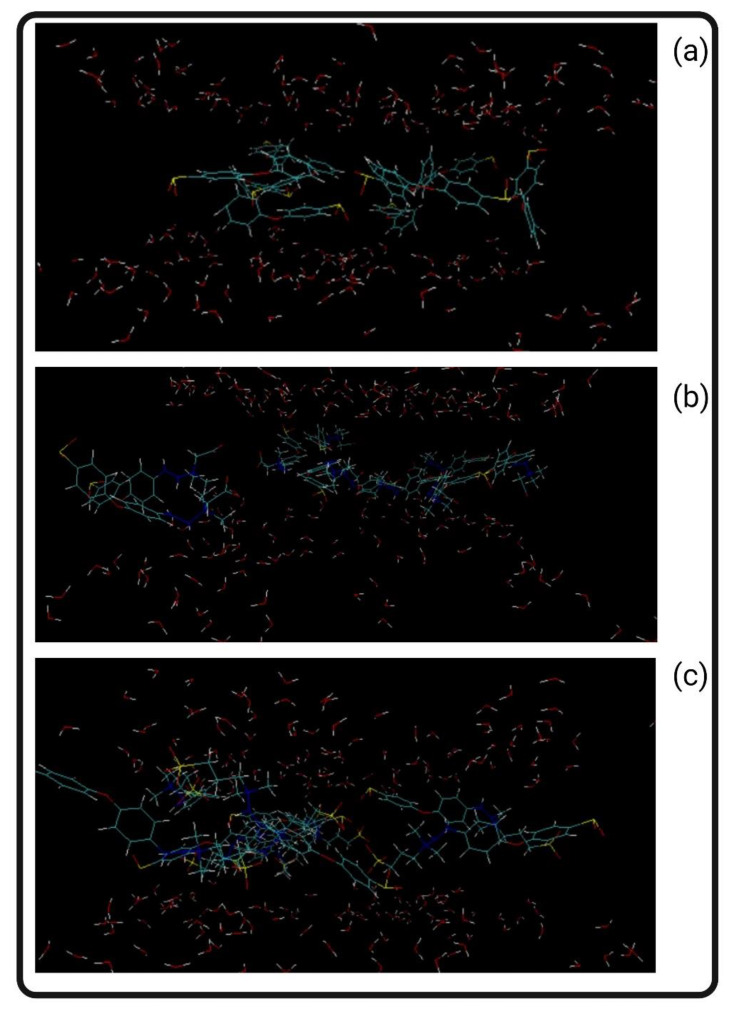
Mobility simulations: (**a**) PES, (**b**) PES-CB, and (**c**) PES-SB.

Figure 13 reflects the simulation captures of the neat and modified membrane models with the surrounding water molecules. Table 12 reflects the results of the mobility simulation. The relative mobility value reflects the reduction of the mobility values for the PES-CB and PES-CB in comparison with the PES structure. The PES-CB system has 17% less mobility, and the PES-SB system has 22% less mobility in comparison with PES.

### 3.11. Induction of Hemocompatibility Assessment and Its Stability

This section is assessing the hemocompatibility and the inflammatory biomarkers released in a patient’s uremic serum when interacting with newly coated membranes compared to the untreated one; in addition, we have evaluated the hemocompatibility of treated membranes after 10 days to evaluate the stability of the hemocompatibility improvement.

**Figure 14:**
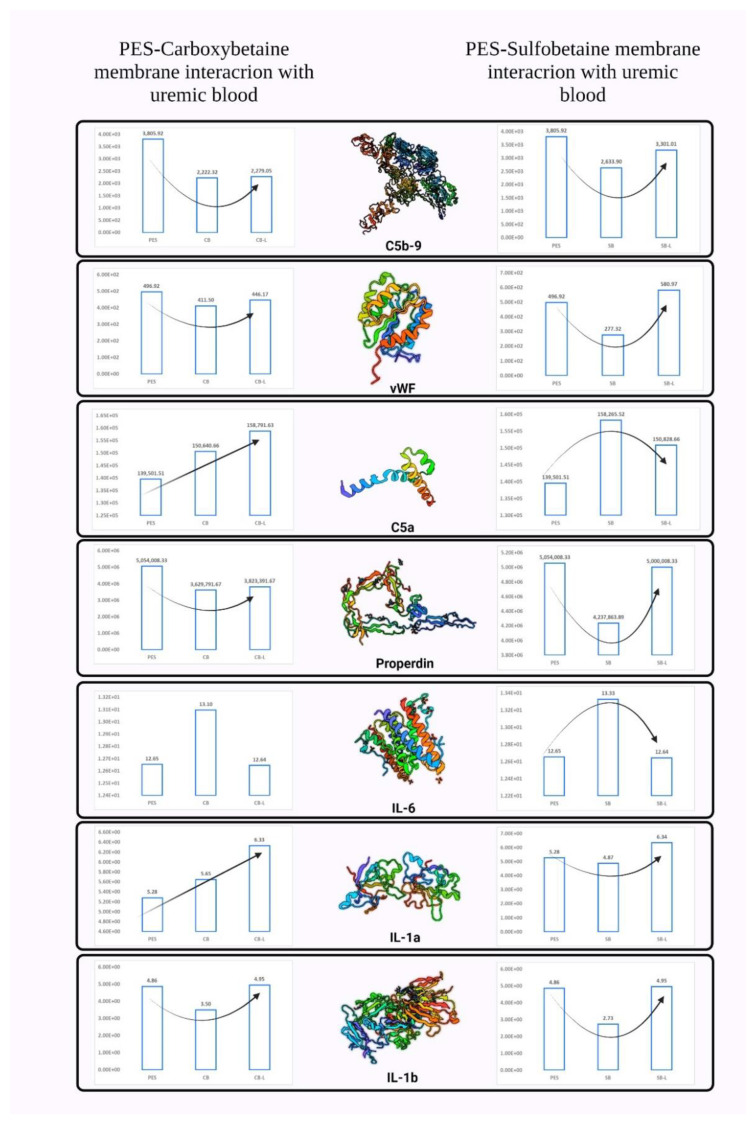
Inflammatory biomarkers released in the patient’s uremic serum after membrane samples incubation (n = 5) (all the concentrations are in pg/mL).

Figure 14 and Table 13 reflect the clinical study results for hemocompatibility in terms of percentage change and the concentration in pg, respectively. Our measurements reflected a lower level of C5b-9 (71% less for PES-CB in comparison with PES). PES-SB resulted in a 44% decrease in the secreted concentration of C5b-9. Plasma terminal C complex C5b-9 complex is a stable and reliable marker of biocompatibility; measuring the concentration of this cytokine could reflect the hemocompatibility extent of the membrane regarding the complement cascade [97]. The enhanced secretion of C5b-9 is due to the limited activation of the complement cascade. Another potential property of the ZW-modified membrane that will support a lower level of C5b-9 production is the stable hydration layer. Since a protected water layer is formed on the PES-CB membrane, the attachment of macromolecules as the initiation mechanism of C5b-9 is prohibited. The stability tests resulted in the growth of the C5b-9 factor to 2.5% for PES-CB-L in comparison with PES-CB and 25.5% for PES-SB-L in comparison with PES-SB.

Cytokines, such as interleukin-1β (IL-1β), tumor necrosis factor-α (TNF-α), and IL-6, may induce an inflammatory state and are believed to play a significant role in dialysis-related morbidity [98]. It has been proven that the basal expression of IL-6 and TNF-α is not a function of the membrane’s hemocompatibility, since they are more affected by the endotoxin content of the dialysate [99]. Accordingly, the variations in the measured IL-6 inflammatory biomarker might not be considered a concern for the loss of hemocompatibility. The two cytokines which were negatively affected by the zwitterionization are IL-1 and IL-6. Commonly the trend of cytokine level change is similar when the blood touches an incompatible membrane [100]. However, we do not see a similar trend between C5a and IL-1 in our measurements. The content of the IL-1a is increased in the PES-CB membrane (6.9% more in comparison with the control sample) while the PES-SB resulted in a 7.7% lower amount of the same interleukin. This could be due to the rich carboxyl and hydroxyl content of PES-CB (similar to the increased content of interleukins in blood-cellulose membrane contact [101]). The PES-CB-L and PES-SB-L resulted in a 12% and 30% increase in the secretion content of IL-1a, respectively.

The trend for IL-1b was similar for both PES-CB and PES-SB samples (a 28% and 43% decrease, in comparison with the control sample). The stability test resulted in the loss of hemocompatibility for the same inflammatory factor to the extent of 41% and 81% for PES-CB-L and PES-SB-L in comparison with PES-CB and PES-SB, respectively. Neither of the PES zwitterionization approaches was effective in reducing the amount of IL-6. The content of IL-6 grew by 3.5% and 5.3% for PES-CB and PES-SB. After keeping the samples in deionized water, the secretion level of IL-6 reached back to 12.6 pg for both PES-CB-L and PES-SB-L (*p* < 0.01). By considering the content of IL-6 secretion, the stability test reflected the loss of hemocompatibility to 3.4% for PES-CB-L and 5.1% for PES-SB-L extents in comparison with PES-CB and PES-SB samples.

Proteins’ adsorption tendency to the dialysis membrane is mentioned to play a role in the release of inflammatory cytokines and vWF [102,103]. The exception to this general rule is C5a and IL-1b [104]. Human C5a anaphylatoxin is a bioactive biomarker that has both spasmogenic and leukocyte-related characteristics. Various properties of C5a make it a critical component for the normal host defense mechanism of the human body. However, the increased level of the biomarker in dialysis sessions could promote the well-known complications of hemodialysis [105]. It is proven that the generation of the complement anaphylatoxin C5a could lead to the expression of active tissue factor (TF) in ESRD. This will contribute to hemodialysis-induced thrombogenesis [106]. The modified PES-CB membrane showed a higher C5a level (7.9% in comparison with the PES control sample). PES-SB membranes even resulted in a higher content of C5a in the blood (13.9%). Previous studies on PES membranes modified with polyvinyl pyrrolidone (PVP) reflect the same results. When the membrane is more negatively charged or when the membrane is rougher, more incompatible interactions are reported [107]. The increased level of C5a for the zwitterionized membranes here could be due to more negatively charged surfaces. This agrees with our zeta potential measurements, as the PES-SB membrane was more negatively charged in comparison with PES-CB. The stability tests reflect that the content of C5a increased for PES-CB-L to 5.4% in comparison with PES-CB. PES-SB-L However, it had a decline in C5a content (4.7%) in comparison with PES-SB.

vWF is a glycoprotein taking part in hemostasis and an identifier for endothelial cell stimulation [108]. Based on the literature, a pronounced inflammatory level of cytokines is mentioned to affect the increase of vWF [109]. Our measurements reflected different patterns for C5a and vWF. We observed a lower concentration of vWF for the modified PES-CB (17% lower than PES) and PES-SB (43% lower than PES), while zwitterionization did not enhance the production of C5a, as discussed before. This does not agree with the exception made for C5a and vWF earlier [104]. vWF is either created in endothelial structures and megakaryocytes or through the granules of platelets [110]. Accordingly, vWF is a known predictor of cardiovascular shocks [111] due to its “complement-thrombogenesis linking nature”. Since the level of C5a was not satisfactory for the PES-CB, the desired modified level of vWF could probably be due to the control of platelet activation. The different patterns of vWF and C5a for PES and PES-CB reflect that inflammation as a result of complement might not necessarily boost the level of vWF. The correlation between the adsorption of plasma proteins such as fibrinogen to the membrane and the amount of vWF has been mentioned elsewhere [112]. The stability tests reflected an 8.4% and 109.4% increase in the secretion of vWF for PES-CB-L and PES-SB-L, in comparison with PES-CB and PES-SB, respectively.

The increase in the concentration level of properdin is linked to the prevalence of neutrophil leukocytes [113]. An increased value was reported in the bloodstream in post-dialysis inflammatory biomarker measurements (the same trend for C5b-9 and C3a [114,115]). Accordingly, blood membrane interactions that lead to the degranulation of polymorphonuclear leukocytes could increase the level of properdin after the dialysis session [116]. PES-CB resulted in a lower concentration of properdin (28% less in comparison with the control sample). The drop in the percentage of properdin was also observed for PES-SB (16% less in comparison with the control sample), which may be attributed to the modified PES-CB membrane provoking the neutrophils in the blood to a lower extent. The stability test reflects that the properdin level for PES-CB-L increased in comparison with PES-CB (5.3%), but the concentration of properdin was still lower in comparison with the PES control sample. PES-SB-L had a higher level of properdin in comparison with PES-SB as well (17.98%).

A comparison between the new approach of ZW implementation and the more known chemical structure of PVP could reflect on how zwitterions. PVP takes advantage of a heterocycle of carbon–nitrogen with double-bonded oxygen. The structure takes advantage of similarity to zwitterions with the two atoms and is negatively charged. Accordingly, the well-known hemocompatible agent is like the structures here. Using the same clinical framework of hemocompatibility assessment, Abdelrasoul et al. reported the extent of biomarker activations for the PES membranes dip-coated in PVP solution from 1 to 4 min [107]. The modification resulted in improvements in C5b-9, IL-1β, IL-6, and Serpin. A higher coating time of PVP (3 and 4 min) led to improvements in C5a activation as well. Similar trends in terms of C5b-9 elimination were observed with the ZW modified membranes. Two out of the three interleukins were triggered by the ZW structures. IL-6 improved with PVP and not ZW structures, which means lower immune system provocation and inflammation could be expected from PVP coated membranes. In terms of complement control, PVP had better performance in comparison with ZW as it could reduce both C5a (which is a primary inflammatory mediator) and C5b-9 (which could result in cell lysis). ZW, on the other hand, could only lower C5b-9. The paper did not offer an analysis of other biomarkers studied in this research, i.e., properdin and vWF. A more generic comparison with more clinical aspects of hemocompatibility is required for a more comprehensive assessment.

## 4. Discussion and Conclusions

This study involved aminolysis-assisted zwitterionization of PES membrane as the most common hemodialysis membrane material. The study covered step-by-step characterization of the synthesized membranes, surface charge measurements, surface roughness pattern investigation, and clinical (in vitro inflammatory biomarker) assessments. The advantage of this modification approach lies in the fast and facile implementation of the functional groups. Aminolysis was chosen instead of harsh amine acid washing-based functionalization. The amine loading could be optimized further by changing the amine monomer type and concentration, as well as the reaction time. Similar optimization is applicable for the negative functional moiety. Accordingly, despite its simplicity, the approach is a perfect fit for commercial production lines. The research offered here was conducted with a minimum number of prepared samples due to the experimental and clinical limitations. A better practice to take another step toward finding the best strategy to implement the proposed modification includes a more rational experimental design to cover a wider range of reaction conditions. This could depend on many aspects of the production procedure as well. The durability of the modification was checked after 10 days, yet a production plan and inventory management system might not allow the use of membrane modules after such a short period of time. Accordingly, a more realistic approach would be to consider the durability of the zwitterionziation after a longer period when designing the experiments.

The ATR-FTIR and XPS characterization techniques proved the existence of quaternary ammonium as well as negative functional groups on the top of the membrane surface. The surface charge measurements through zeta potential revealed the negatively charged surface of both zwitterionized structures, which means we located more carboxyl and sulfonate than ammonium in the layer-by-layer assembly of the zwitterionic structures. One of the concerns is regarding the control over the electroneutrality of the ZW structure. By definition, a ZW structure is an electroneutral combination of a positive and negative charge, yet the reports reflect that the overall moiety could be far away from the inert state due to the polymerization reaction conditions.

In vitro studies of the inflammatory biomarkers reflected a better hemocompatibility profile between the modified membranes, specifically PES-SB. Inflammatory cytokines are signaling interleukins that could stimulate cancer cell growth, take part in locoregional relapse, as well as metastasis, induce cardiovascular shocks, activate C-reactive proteins, and result in inflammation. IL-1 induces the production of IL-6. We obtained satisfactory results from IL1-b. We had a higher level of IL-1 and IL-6 after zwitterionziation. The complement cascade, as a part of the innate immune system, contributes to the defensive activities of the body against infections. A hemocompatible membrane would preferably lower the stimulation of the immune system through lower secretion of complement factors C3a, C5a, and C5b-9. The lower level of C5b-9 alongside the higher amount of C5a reflects a partially enhanced level of hemocompatibility for our zwitterionized membranes. We also have satisfactory results regarding properdin’s secretion decrease, which means we could successfully control the complement activation through the alternative pathway. vWF is believed to be induced by the increased amount of C5a. However, on the membrane surface, there had to be other factors affecting the lower level of vWF, such as the hydration layer. Accordingly, the modified membranes have a higher chance of hemocompatibility regarding lower cardiovascular-complement complications out of a pronounced vWF level. It is important to note that hemocompatibility could be assessed from different perspectives. The data offered in this research were based on the inflammatory biomarkers released in the blood when they were incubated with blood. However, we did not report the clotting times or platelet activation enzyme concentrations. More investigations need to be conducted to obtain an in-depth understanding of the current membranes’ hemocompatibility.

Our stability test reflected that a portion of surface charge (and accordingly a part of the zwitterionic structure) was lost when the membranes were kept in deionized water for 10 days. While amine functionalization as well as both ziwtterionization methodologies have been successfully tested previously, the instability of the quaternary ammonium is still a concern. It has been reported that the quaternary ammonium could be unstable, depending on the chemistry of the surrounding environment and the structures. Another concern would be the stability of the structures under real dialysis filtration conditions when shear stress could erode the surface of the membranes. Accordingly, the charge loss and the degradation of the hemocompatibility degree of the overall ZW structure have to be further investigated.

The stability of water has been identified as the main reason for the hemocompatibility of the dialysis membrane, rather than its hydrophilicity. The hypothesis works as a material science shortcut for the clinical methodology (coagulation, clotting time, complement, and inflammation cascades). This could be accurate to a great extent, as the stable hydration layer could eliminate the interaction of the blood constituents with the membrane surface. However, it does not cancel the need for clinical investigations for a membrane product. The IR-based spectroscopy here reflected stronger hydrogen bonds between the modified membranes in comparison with the control sample. EWC calculations reflected that the modified membranes are capable of keeping hydration layers on and within the porous membrane structure as well.

The AFM-assisted roughness study reflected that the modified membranes had smoother surfaces, and accordingly, they had a lower chance of protein interaction and fouling in general. The smoother surface of the modified membranes also reflects the higher hemocompatibility of the products, due to lower protein fouling and lower chances of rupturing the blood cells (less hemolytic surfaces).

The approach introduced in this study covers fast and facile zwitterionization (CB and SB structures) on common hemodialysis membrane materials. The same extent of surface charge loss was observed for both membranes, while PES-CB had a slightly smoother surface and, accordingly, a lower chance of protein fouling. While the response of PES-SB to IL-1a was better than that of PES-CB, the rest of the inflammatory biomarkers and cytokines reflected a similar trend. Accordingly, both modified membranes showed more hemocompatibility, considering their clinical response to uremic blood samples and their surface morphology profile.

## Figures and Tables

**Table 1:** Bond parameters.

Bonds	r_0_ (Å)
C-C	1.53
N-N	1.022
O-S	1.69
C-H	0.98
N-C	1.46
O-C	1.42
S-C	1.8
N-H	1.02
O-H	0.98

**Table 2:** Angle parameters.

Central Atom	Angle θ_0_ (Degree)
Carbon	109.47
Hydrogen	180
Nitrogen	106.7
Sulfur	92.1
Oxygen	104.51

**Table 3:** Non-bond parameters.

Atom	ε (kcal/mol)	σ (Å)
C	0.0238	3.473
H	0.0038	2.846
N	0.0194	3.263
O	0.0239	3.033
S	0.0860	3.590

**Table 4:** Chemical structures of neat and modified PES for the computational simulations.

	Name	Structure
1	Polyethersulfone (PES)	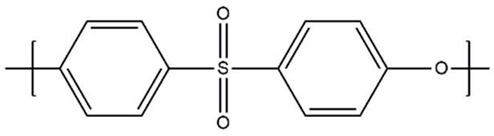
2	Polyethersulfone-carboxybetaine (PES-CB)	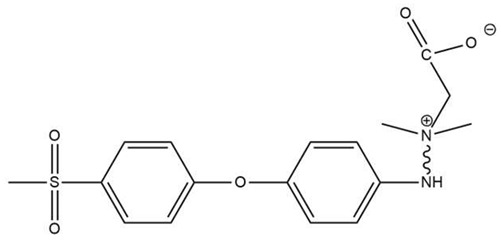
3	Polyethersulfone-sulfobetaine (PES-SB)	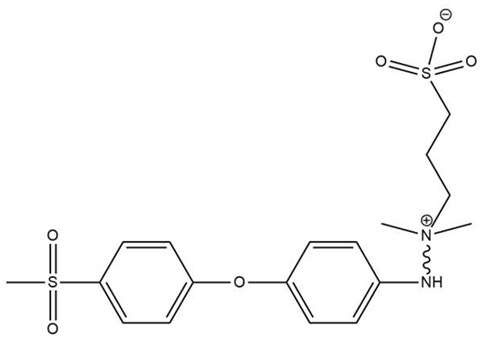

**Table 5:** Simulation details of the hydrogen bonding and mobility simulations.

System	Simulation	Number of Polymers	Number of Water Molecules	Number of Overall Atoms in the Simulation
PES	Hydrogen bonding	1	60	205
PES-CB	Hydrogen bonding	1	60	223
PES-SB	Hydrogen bonding	1	60	230
PES	Mobility	10	200	850
PES-CB	Mobility	10	200	1030
PES-SB	Mobility	10	200	1100

**Table 6:** Elemental percentage analysis of the neat and modified membranes using XPS spectroscopy.

Sample Name	Carbon (%)	Oxygen (%)	Nitrogen (%)	Sulfur (%)
PES	72.78	20.41	0.92	5.90
PES-NH2	75.27	15.26	4.26	5.21
PES-SB	73.46	15.19	4.74	6.61
PES-CB	75.39	16.54	3.18	4.90

**Table 7:** Grafting amount measurement after zwitterionization of PES membrane with CB and SB.

Membrane	GA (mg)	STD	Error (%)
PES-SB	0.27	0.12	±0.07
PES-CB	0.23	0.15	±0.09

**Table 8:** The equilibrium water content of neat and zwitterionized PES membranes.

Membrane	EWC (%)	STD	Error (%)
PES	43.13	2.98	±1.72
PES-CB	49.81	3.81	±2.2
PES-SB	52.27	5.22	±3.01

**Table 9:** The stable water percentage for PES, PES-CB, and PES-SB membranes.

Membrane	Non-Freezable Water (%)
PES	2.83
PES-CB	12.85
PES-SB	16.28

**Table 10:** Surface area, pore volume, and pore size of the membranes, device precision ± 0.1 %.

**Membranes**	**BET-Specific Surface Area (m^2^/g)**	**Pore Volume (cm^3^/g)**	**Pore Diameter Range** **(nm)**
PES	78.10	0.06	More than 50
PES-CB	93.50	0.07	2–50
PES-SB	89.60	0.16	2–50

**Table 11:** Comparison of hydrogen bonding between hydrated PES and PES-CB and PES-SB systems.

System	Energy of Hydrogen Bonding (kcal/mol)	Number of Hydrogen Bonding	E/n (kcal/mol per Bond)
PES	−14.37	3	−4.79
PES-CB	−1.45	11	−0.13182
PES-SB	−0.03	2	−0.015

**Table 12:** Mobility values for PES, PES-CB, and PES-SB membranes.

System	Mobility Value (Unitless)	RMV	RMV (%)
PES	6.96 × 10^−6^	1.00 × 10^0^	100.00
PES-CB	5.74 × 10^−6^	8.25 × 10^−1^	82.47
PES-SB	5.49 × 10^−6^	7.89 × 10^−1^	78.88

**Table 13:** Inflammatory biomarkers in vitro clinical measurement; comparison of modified membrane responses with the neat PES membrane (n = 5).

Membrane	C5a [pg/mL]	IL-1β [pg/mL]	IL-1α [pg/mL]	IL-6 [pg/mL]	vWF [pg/mL]	Properdin [pg/mL]	C5b-9 [pg/mL]
PES	139,501.51	4.86	5.28	12.65	496.92	5,054,008.33	3805.92
CB	150,640.66	3.50	5.65	13.10	421.50	3,829,791.67	2722.32
CB-L	158,791.63	4.95	6.33	12.64	446.17	3,823,391.67	2779.05
SB	158,265.52	2.73	4.87	13.33	580.97	4,237,863.89	2633.90
SB-L	150,828.66	4.95	6.34	12.64	277.32	5,000,008.33	3301.01

## Data Availability

The raw/processed molecular docking data required to reproduce these findings cannot be shared at this time, as they are critical to ongoing research.

## List of Abbreviations

**Name**	**Abbreviation**
Atomic force microscopy	AFM
Attuned total reflectance–Fourier transmission infrared	ATR-FTIR
Brunauer–Emmett–Teller	BET
Differential scanning calorimetry	DSC
Diethyl triamine	DETA
End-stage renal disease	ESRD
Equilibrium water content	EWC
Interleukin	IL
Molecular dynamics	MD
Phosphobetaine	PB
Polyethersulfone	PES
Polysulfone	PSF
Polyvinyl pyrrolidone	PVP
Polyvinylidene fluoride	PVDF
Polyethylene-co-vinyl alcohol	EVOL
Polylactic acid	PLA
Polyvinyl chloride	PVC
Picograms per milliliter	pg/mL
Root mean square	RMS
Sodium polystyrene sulfonate	PSSNa
Sulfobetaine	SB
Surface area	SA
Tumor necrosis factor	TNF
Von Willebrand factor	vWF
X-ray photoelectron spectroscopy	XPS
Zwitterionic	ZW
